# Slower adaptation of control strategies in individuals with high impulsive tendencies

**DOI:** 10.1038/s41598-021-99764-1

**Published:** 2021-10-13

**Authors:** Fanny Grisetto, Yvonne N. Delevoye-Turrell, Clémence Roger

**Affiliations:** Univ. Lille, CNRS, UMR 9193 - SCALab - Sciences Cognitives et Sciences Affectives, F-59000 Lille, France

**Keywords:** Human behaviour, Psychiatric disorders

## Abstract

Flexible use of reactive and proactive control according to environmental demands is the key to adaptive behavior. In this study, forty-eight adults performed ten blocks of an AX-CPT task to reveal the strength of proactive control by the calculation of the proactive behavioral index (PBI). They also filled out the UPPS questionnaire to assess their impulsiveness. The median-split method based on the global UPPS score distribution was used to categorize participants as having high (HI) or low (LI) impulsiveness traits. The analyses revealed that the PBI was negatively correlated with the UPPS scores, suggesting that the higher is the impulsiveness, the weaker the dominance of proactive control processes. We showed, at an individual level, that the PBI increased across blocks and suggested that this effect was due to a smaller decrease in reactive control processes. Notably, the PBI increase was slower in the HI group than in the LI group. Moreover, participants who did not adapt to task demands were all characterized as high impulsive. Overall, the current study demonstrates that (1) impulsiveness is associated with less dominant proactive control due to (2) slower adaptation to task demands (3) driven by a stronger reliance on reactive processes. These findings are discussed in regards to pathological populations.

## Introduction

Driving is a complex behavior that requires efficient attentional and executive functions (e.g., inhibition, updating, working memory) to execute the appropriate action to stay adapted in a constantly changing and unpredictable environment. Imagine arriving in a crowded area where the visibility is low. As a driver, to avoid an accident, you face two choices. You can either wait and react with an emergency braking if something unexpected happens (e.g., a pedestrian crosses) or you can anticipate an event by slowing down the speed of the car. This choice is made as a function of the context (e.g., other cars in the street or not) and of inter-individual differences. These daily situations require cognitive control processes to resolve the conflict (i.e., co-activation of responses). The current study aimed at uncovering inter-individual differences in the implementation of cognitive control strategies.

Cognitive control is the ability to adjust goal-directed behaviors according to internal goals and external demands, supported by basic executive functions^1,2^. Two distinct control strategies are involved in conflict resolution^3,4^. On the one hand, reactive control corresponds to a transient, stimulus-driven form of cognitive control (e.g., emergency brake). On the other hand, proactive control reflects a sustained, anticipatory form of cognitive control (e.g., slowing down). In the dual mechanisms of control (DMC) framework^4^, proactive and reactive mechanisms co-exist in the cognitive control system as two opposite poles of a continuum. Their involvement in conflict resolution relies on a tradeoff between the benefits and the costs of proactive and reactive strategies according to the current situation^4^. However, it is also crucial to investigate which and how inter-individual differences affect control strategies to understand differences in behavioral tendency.

In line with the DMC framework, the AX-variant of the Continuous Performance Task paradigm (i.e., AX-CPT) was developed to measure goal representation, maintenance and updating^5,6^. In this paradigm, the participant is required to respond “yes” when he/she sees an “X” following an “A”, and to respond “no” whenever another letter combination is presented. The manipulation of the expectancy of the cue letter “A” and the probe letter “X” (i.e., AX in 70% of the trials) creates two distinct conflictual situations. In AY trials, the participant expects to see an “X” after the “A” and must inhibit the prepotent response when facing a non-X letter, represented by a Y. In BX trials, the “X” probe triggers an automatic response that must be inhibited since the cue is a non-A letter, represented by a B. The difference between mean reaction times (RTs) in AX trials and in AY trials on the one hand, and between RTs in AX and in BX trials on the other hand, reveal two different conflict costs. The difference between these two types of conflict (i.e., mean RTs in AY trials and in BX trials) reflects the dominancy between the two control mechanisms. When proactive control mechanisms are dominant, RTs are longer in AY trials than in BX trials, indicating that the participant uses the cue letter to prepare for action. On the contrary, when reactive control mechanisms are dominant, RTs are longer in BX trials than in AY trials, revealing that the participant does not process correctly the cue and waits for the probe before retrieving the goal representation on which to base his/her answer. The proactive behavioral index (PBI) reflects this difference and can be used to estimate objectively the relative strength of the engagement of proactive control mechanisms over reactive ones^5^.

In young healthy adults, RTs in AY trials are often reported to be longer than those in BX trials, suggesting the predominant use of proactive control processes^7–10^. Using predictable and unpredictable environments to reveal the dynamics of proactive control, a recent study showed that proactive control processes are already set at trial start suggesting that this mechanism is the default state of cognitive control^11^. Nevertheless, this default state varies as a function of inter-individual differences^5,12^ such as age^13^ and working memory capacity^14,15^. However, to our knowledge, little is known about the effect of personality traits on the default state of cognitive control. In pathological populations, the AX-CPT performances were compared between attention deficit hyperactivity disorders (ADHD), borderline personality disorder (BPD) patients and healthy controls^7^. In ADHD patients, although the pattern of results was similar to that observed in healthy controls (i.e., longer RTs in AY trials than in BX trials), the AY-BX difference was smaller in these patients compared to that observed in healthy controls. On the contrary, in BPD patients, the RTs were longer in the BX than in the AY trials. Overall, these findings indicated that the dominance of proactive strategies, as the default state observed in healthy controls, was less pronounced in ADHD and could even be shifted towards reactive control in BPD (i.e., negative AY-BX difference). Interestingly, both of these pathologies have been largely characterized by impulsive behaviors^16,17^. According to these previous findings, we hypothesized that in the general population, individuals with high impulsive traits would adopt lower proactive control strategies compared to individuals with low impulsive traits (H1). Impulsivity was here globally assessed through the total score of the UPPS questionnaire^18^.

**Figure 1:**
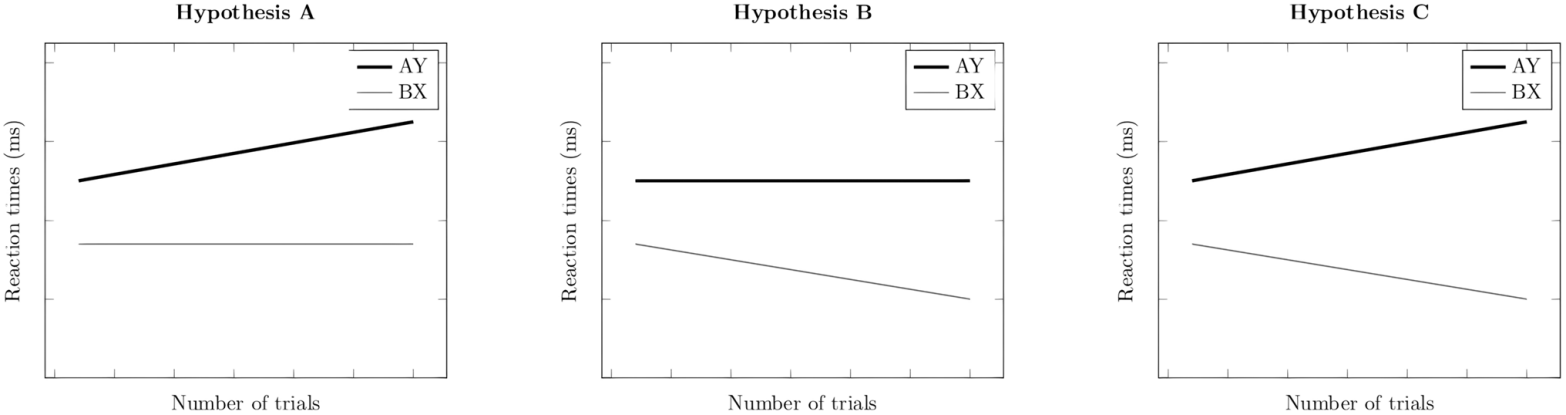
Graphical representations of the three alternatives to explain the increase in AY—BX difference with the increase in the number of trials. Greater proactive behavioral index in studies with larger number of trials could be guided by three alternatives: the increase in proactive control reflected in the progressive increase of AY trials RTs (**A**), the decrease in reactive control reflected in the progressive decrease in RTs in BX trials (**B**), or the combination of both patterns (**C**).

**Figure 2:**
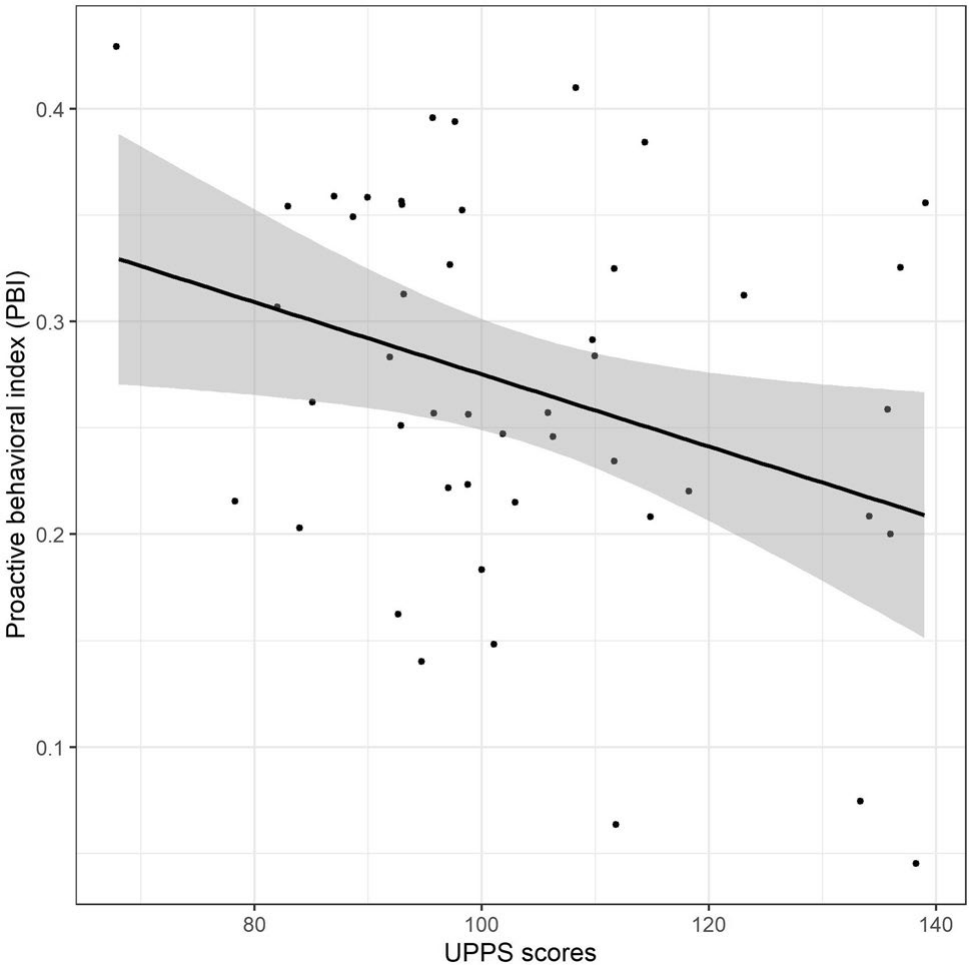
Correlation between the impulsiveness scores, assessed through the UPPS scores, and the global PBI calculated across all blocks. The grey area represents the 0.95 confidence interval band.

**Figure 3:**
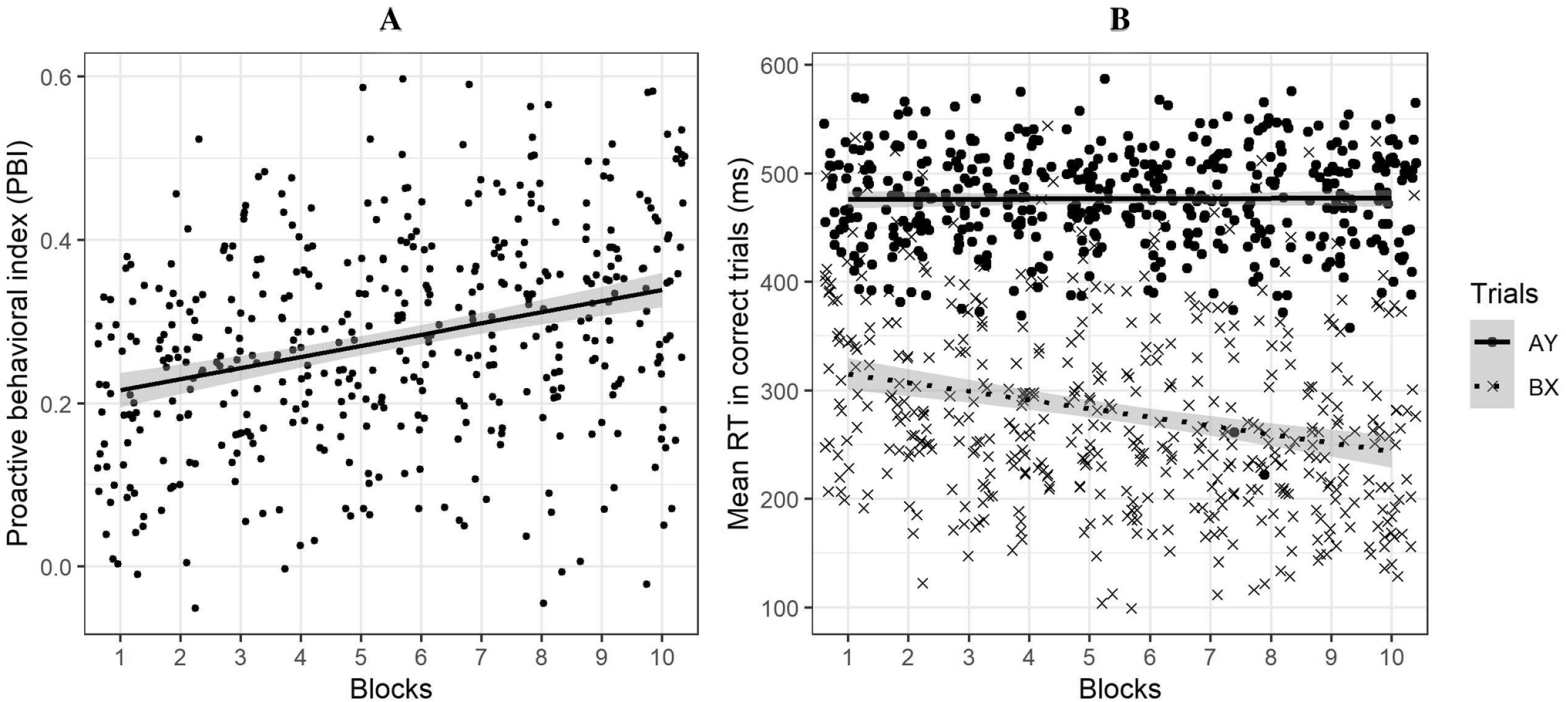
Evolution of the proactive behavioral index (**A**) and the RTs in correct AY and BX trials (**B**) across blocks. (**A**) The proactive behavioral index (PBI), calculated as (AY − BX)/(AY + BX), increased across blocks. (**B**) The BX trials RTs progressively decreased whereas the AY trials RTs remained stable across blocks. The increase in the PBI is therefore due to a decrease in the involvement of reactive control processes. The grey areas represent the 0.95 confidence interval bands.

The default state may change as the environmental demands change. Indeed, a recent meta-analysis showed that the AY-BX difference in RTs increased in studies with a large number of trials^19^, suggesting the possibility of a gradual adaptation of proactive processes over time in the AX-CPT task. To our knowledge, this hypothesis has not been investigated at the individual level so far (nor its potential modulation by inter-individual differences). If the adaptation over time is confirmed, then three possibilities can be considered to explain the increase in the AY-BX difference: (1) an increase in RTs in AY trials, reflecting a growing involvement of proactive processes over time (Fig. 1A), (2) a decrease in RTs in BX trials, reflecting a weakening of reactive processes over time (Fig. 1B) or (3) the combination of the two possibilities (Fig. 1C). Thus, dissecting the increase in proactive control over time will offer a deeper understanding of the adaptative nature of the cognitive strategies over time. The current study aimed at identifying which one of the three patterns could explain the shift towards a greater proactive control dominance observed in the normal adult population, while investigating the influence of impulsive personality traits on the adaptation ability of control strategies (H2).

## Results

### Global analyses

In order to assess the effects of trial type on accuracy and reaction times in correct trials, we computed two one-way ANOVAs. Results revealed a main effect of Trial Type on RTs in correct trials and on accuracy, *F* (3, 138) = 503.1, *p* < 0.001, *η*^2^ = 0.92, Bayes Factor (BF_10_) = 1.09^e71^ and *F* (3, 138) = 43.91, *p* < 0.001, *η*^2^ = 0.49, BF_10_ = 1.83^e71^, respectively (cf. Table 1). Looking at the confidence intervals, we observed that participants were slower and less accurate in AY trials than in other trial types revealing the dominant use of proactive strategy during the task. Accordingly, the mean proactive behavioral index (PBI) calculated with the RTs in correct AY and BX trials across all blocks was 0.27 (95% CI [0.24, 0.30]).

**Table 1:** Means and 95% Confidence Intervals in Global and By-Trials Accuracy (%) and Reaction Times in Correct Trials (RT, ms), and the Proactive Behavioral Index (PBI) in Low (LI) and High (HI) Impulsiveness Groups.

	Total (n = 47)		LI (n = 22)		HI (n = 23)	
	M	95% CI	M	95% CI	M	95% CI
Total accuracy (%)	90.39	[88.89, 91.90]	90.59	[88.57, 92.61]	89.70	[87.31, 92.10]
AX accuracy (%)	96.98	[96.30, 97.66]	97.17	[96.25, 98.09]	96.64	[95.53, 97.74]
AY accuracy (%)	81.06	[76.82, 85.30]	80.71	[75.63, 85.80]	80.37	[72.91, 87.83]
BX accuracy (%)	91.12	[89.05, 93.20]	91.23	[88.22, 94.24]	90.62	[87.31, 93.93]
BY accuracy (%)	92.40	[90.49, 94.32]	93.25	[90.45, 96.05]	91.18	[88.24, 94.12]
Global RT (ms)	347.61	[333.48, 361.73]	335.99	[314.50, 357.48]	358.50	[338.58, 378.41]
RT in AX (ms)	344.86	[331.16, 358.57]	338.46	[320.04, 356.87]	350.27	[327.64, 372.89]
RT in AY (ms)	477.82	[466.82, 488.82]	479.23	[464.63, 493.84]	477.67	[458.95, 496.39]
RT in BX (ms)	281.68	[260.70, 302.66]	261.45	[235.15, 287.75]	300.78	[265.86, 335.71]
RT in BY (ms)	286.07	[265.65, 306.48]	264.84	[238.01, 291.67]	305.27	[272.45, 338.09]
PBI	0.27	[0.24, 0.30]	0.30	[0.27, 0.34]	0.24	[0.20, 0.28]

### Effect of impulsiveness on the PBI

Accuracy rates, reaction times (RT) and proactive behavioral index (PBI) were compared between the low and the high impulsiveness groups (LI and HI, respectively) using Student’s *t*-test. Data are presented in Table 1. The HI and LI groups did not differ on global accuracy rates, *t*(174.28) = 0.56, *p* = 0.574, Cohen’s *d* = 0.08, BF_10_ = 0.19, or on global RTs, *t*(176.32) = 1.53, *p* = 0.129, Cohen’s *d* = 0.23, BF_10_ = 0.48. There was a main effect of Impulsiveness on the global PBI, *t*(42.09) = 2.42, *p* = 0.020, Cohen’s *d* = 0.72 (medium effect), BF_10_ = 2.86. The PBI was smaller in the HI group (*M* = 0.24, 95% CI [0.20, 0.28]) than in the LI group (*M* = 0.30, 95% CI [0.27, 0.34]).

More specifically, the current study revealed a negative correlation between impulsiveness scores and the PBI, *r* = − 0.33, *p* = 0.026, BF_10_ = 3.04: the higher the UPPS scores, the smaller the PBI (cf. Fig. 2). Investigating the four dimensions of the UPPS questionnaire, results showed that the PBI was correlated negatively with premeditation and sensation seeking scales, *r* = − 0.31, *p* = 0.031, BF_10_ = 2.61 and *r* = − 0.29, *p* = 0.045, BF_10_ = 1.98, respectively. However, the PBI did not correlate with the lack of perseveration and the urgency scales, *r* = − 0.18, *p* = 0.222, BF_10_ = 0.64 and *r* = − 0.12, *p* = 0.412, BF_10_ = 0.44, respectively.

### Adaptation of the proactive control strategy across blocks

To explore the evolution of the PBI across the ten blocks, we computed a one-way ANOVA. Results revealed a main effect of Blocks on the PBI, *F* (9, 391) = 14.05, *p* < 0.001, *η*^2^ = 0.24, BF_10_ = 4.04^10^16^. In our entire sample, the PBI increased over time from 0.18 to 0.33 (cf. Fig. 3A). To understand this increase, we investigated the changes in RTs in the AY and BX trials across blocks using a two-way mixed-ANOVA. The model had a BF_10_ of 3.52^e308^ There were significant main effects of Trial Type and Blocks on the RTs, *F* (1, 43) = 604.68, *p* < 0.001, *η*^2^ = 0.93 and *F* (1, 812) = 73.14, *p* < 0.001, *η*^2^ = 0.08, respectively. More importantly, the ANOVA revealed an interaction effect between Trial Type and Blocks on the mean RTs in correct AY and BX trials, *F* (1, 812) = 70.77, *p* < 0.001, *η*^2^ = 0.08 (cf. Fig. 3B). The RTs in the BX trials decreased progressively across blocks whereas the RTs in AY trials remained stable, thus explaining the increase in the AY-BX difference used in the calculation of the PBI.

**Figure 4:**
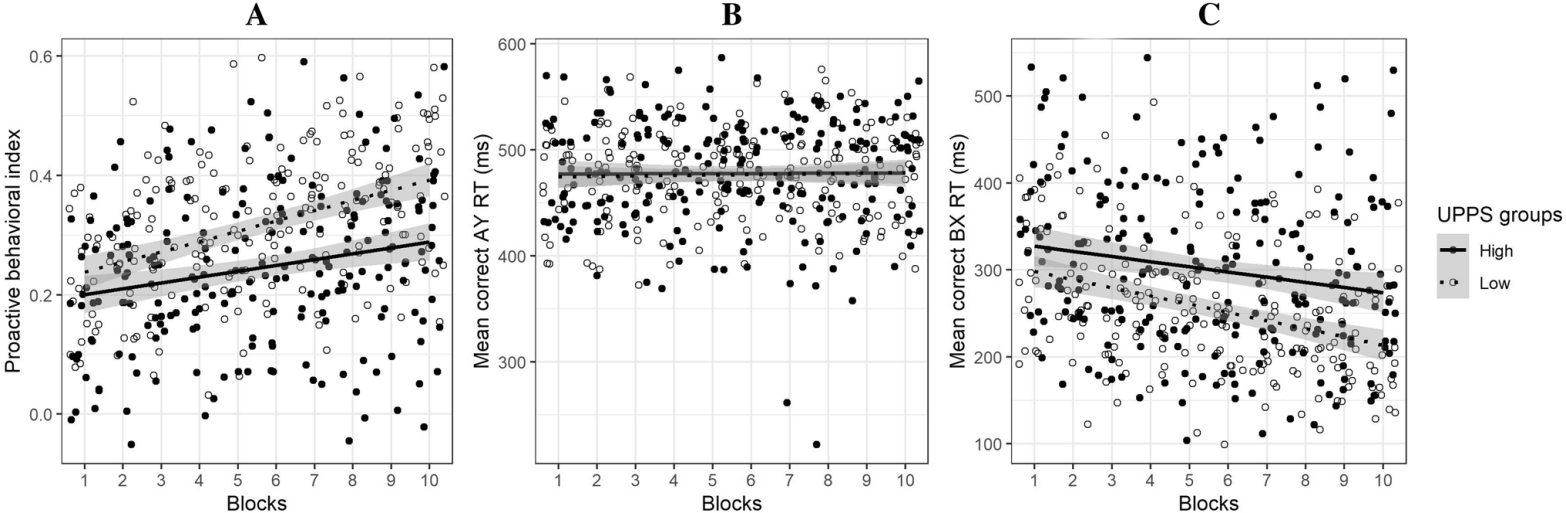
Results obtained in the impulsiveness groups for the PBI (**A**) and for the RTs in AY and BX trials (**B, C**) as a function of blocks. The high and the low impulsiveness groups (filled and empty dots, respectively) were created using the median of the UPPS score distribution. The proactive behavioral index (PBI) in the high impulsiveness group increased slower than the PBI in the low impulsiveness group (**A**). The difference in the PBI adaptation was due to a slower decrease in RTs in the BX trials in the high impulsiveness group compared to that observed in the low impulsiveness group (**C**) whereas RTs in the AY trials remained stable in both groups (**B**). The grey areas represent the 0.95 confidence interval bands.

The effect of impulsiveness on the evolution of the PBI across blocks was investigated using a two-way ANOVA with Blocks as within-subject factor and Impulsiveness as between-subject factor. Results revealed an interaction effect between Impulsiveness and Blocks on the PBI, *F* (1, 42) = 5.73, *p* = 0.021, *η*^2^ = 0.12, BF_10_ = 6.54^10^14^ (cf. Fig. 4A). The PBI increased in both impulsiveness groups, but this increase was slower in the HI group than in the LI group. In the LI group, the PBI increased from 0.22 (95% CI [0.18, 0.27]) in the first block to 0.40 (95% CI [0.35, 0.46], ∆ = 0.18) in the last block whereas, in the HI group, the PBI increased from 0.16 (95% CI [0.11, 0.21]) to 0.28 (95% CI [0.21, 0.34], ∆ = 0.12). In the first block, there were no differences on the PBI between the HI and LI groups, *t*(41) = 1.91, *p* = 0.063, Cohen’s *d* = 0.57 (medium effect). In the last block, the PBI was statistically different between the LI and the HI groups, *t*(40.60) = − 2.99, *p* = 0.005, Cohen’s *d* = 0.60 (medium effect).

Going further to understand the difference in the PBI adaptation between high and low impulsiveness groups, we investigated the changes in the RTs in AY and BX trials across blocks as a function of the impulsiveness groups using a three-way mixed-ANOVA. The full model had a BF_10_ of 5.90^e301^. Firstly, we observed an interaction effect between Trial Type (AY and BX) and Impulsiveness (HI vs. LI), *F* (1, 774) = 51.20, *p* < 0.001, *η*^2^ = 0.06. The HI group had longer RTs in the BX trials compared to that observed in the LI group, but there were no group differences in RTs in the AY trials (cf. Table 1). Therefore, the smaller PBI in the HI group compared to the LI group was explained by longer mean RTs in the BX trials. Secondly, regarding the evolution of the PBI difference through the blocks, we observed a close-to-significant interaction effect between Blocks, Trial Type and Impulsiveness factors, *F* (1, 774) = 3.77, *p* = 0.053, *η*^2^ = 0.005. As there were no differences in RTs in the AY trials across blocks between the HI and the LI groups (see confidence intervals, cf. Fig. 4B), this close-to-significance three-way interaction effect suggested that the RTs in the BX trials decreased more steeply in the LI group throughout the course of the blocks compared to the RTs in the BX trials in the HI group (see confidence intervals, cf. Fig. 4C).

### Estimation of individual adaptation capacities

**Figure 5:**
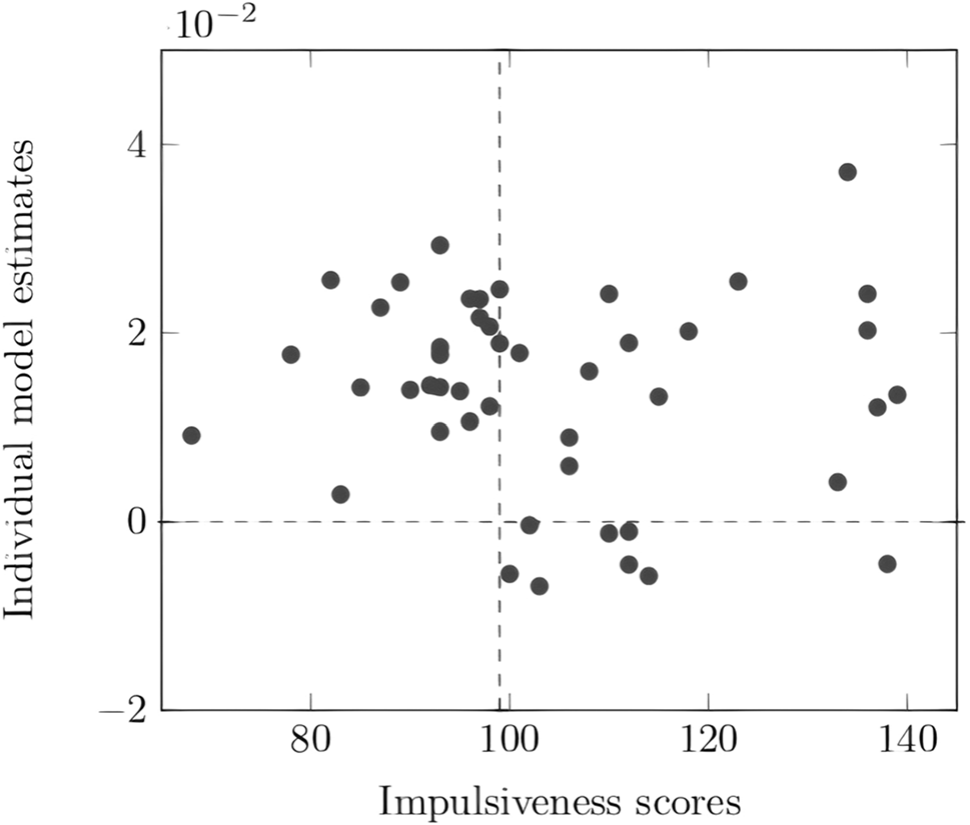
Individual estimates of the model according to the impulsiveness scores. The estimates of the model were used as an index of the capacity to adapt to task demands. The vertical dashed line represents the median of the UPPS score distribution that was used to create the low and the high impulsiveness groups. The horizontal dashed line represents the separation between negative slope (estimate < 0, no adaptation to task demands) and positive slope (estimate > 0, adaptation to task demands).

The individual estimates of the linear regression model were used to investigate possible inter-individual differences in the capacity to adapt spontaneously the control strategies to task demands. These estimates did not correlate with UPPS scores, *r* = − 0.07, *p* = 0.630, BF_10_ = 0.37, nor with any of the four UPPS subscales, all *p* > 0.050, all BF_10_ < 1. However, the *χ*^2^ test revealed a significant association between Impulsiveness groups and the adaptation rate, *χ*^2^ (2, *N* = 44) = 6.74, *p* = 0.009 (cf. Fig. 5). A Bayesian test of association produced a BF_10_ of 31:1 in favor of a relationship between Impulsiveness groups and PBI adaptation. The frequency of observation of a negative estimate was null in the LI group, indicating that LI individuals always adapted their control strategies to the task. However, eight out of 23 individuals in the HI group were characterized by a negative regression model estimate, indicating an absence of adaptation to task demands in 35% of the HI group (cf. Table 2).

**Table 2:** Contingency table between impulsiveness groups and the adaptation of the PBI across blocks as indexed by the model estimates. A positive estimate indicates a global increase in the PBI across blocks, used as an index of adaptation capacities. A negative estimate indicates an absence of evolution or even a decrease in the PBI across blocks and thus, no adaptation to task demands. *LI*: low impulsiveness, *HI*: high impulsiveness.

	LI	HI	Total
Positive estimate	21 (100%)	15 (65%)	36 (82%)
Negative estimate	0 (0%)	8 (35%)	8 (18%)
Total	21	23	44

## Discussion

This study aimed at exploring the effect of impulsivity on the implementation of cognitive control strategies in a healthy population. The AX-CPT paradigm^5,6^ and the UPPS questionnaire^18^ were used to calculate the proactive behavioral index (PBI) and to assess impulsiveness, respectively. Results showed that the high impulsiveness group had a smaller PBI than the low impulsiveness group, suggesting that impulsive individuals relied less on proactive control mechanisms to perform the task than less impulsive individuals. Furthermore, the analysis of the PBI across blocks revealed that participants spontaneously adapted their control strategy to task demands by reducing the involvement of reactive control. Thus, the PBI increased over time, but more slowly in the high impulsiveness group compared to that observed in the low impulsiveness group. This effect might be explained by the eight high impulsive participants that did not adapt control strategies to task demands at all. Overall, the current study demonstrated that high impulsiveness is characterized by a poorer, or even an absent, spontaneous adaptation of control strategies to proactive task demands.

The proactive behavioral index (PBI) is an indicator of relative tendencies towards proactive versus reactive control strategies^5,12^. In the current sample, we found a global positive PBI, consistent with previous findings suggesting that proactive control is the default state of cognitive control^11^. However, in the present study, the high impulsiveness group had a smaller PBI than the low impulsiveness group, suggesting a less dominant proactive control mode in impulsive individuals. The negative correlation between the PBI and the impulsiveness score seems also to suggest that the higher the impulsive tendencies, and more particularly the lack of premeditation and the sensation seeking tendencies, the less dominant is the proactive control. However, the Bayesian statistics indicated only anecdotal or moderate evidences for these effects. Therefore, further studies are needed to confirm the PBI as a potential marker of impulsiveness. Nonetheless, the present findings are consistent with a limited number of results reported in studies investigating impulsive psychiatric populations: the proactive mode is less dominant in schizophrenic and bipolar patients^20^ (calculated PBI = 0.10). It has even been observed that in some specific disorders, such as borderline personality disorder, the default state is reactive control, characterized by a negative PBI^7^ (calculated PBI = − 0.07). However, the relationship between proactive control and impulsivity in pathological populations is still not entirely well defined. If proactive control difficulties were observed in ADHD patients^21^ and in dependent populations^22,23^, the same pattern was not replicated in gamblers^24^ or in another sample of ADHD patients^25,26^. Nevertheless, these later findings do not contradict the results from the current study. Indeed, proactive control was measured at the capacity level in many ways (e.g., ERP components, global RT slowing), but did not assess the relative weight of proactive control strategy compared to reactive control strategy reflected in the PBI. Overall, the current study suggests that the PBI, reflecting the dominance of proactive control strategy, could be a good candidate to objectively measure impulsive tendencies in both normal and pathological populations.

The default state of cognitive control^11^ is not the only parameter of cognitive control that can explain maladaptive behaviors. Indeed, a change in the default state is not dysfunctional if one can efficiently adapt control strategies according to task demands. In the current study, the AX-CPT task was constructed to encourage proactive control processes^19^. Consequently, individuals adapted their control strategies to rely more and more on proactive processes throughout the task, as revealed by the increase in the PBI across blocks. This result was consistent with previous group findings^19^, but we demonstrated the phenomenon at an individual level. Moreover, the current results revealed an inter-individual variability in this adaptation capacity. The increase in the PBI was slower in the high impulsiveness group than in the low impulsiveness group. Therefore, high impulsive individuals were not less proactive per se, but had more difficulty to adapt their control strategy to task demands. Furthermore, the absence of adaptation of control strategy to task demands was observed in high impulsive individuals only (35% of the high impulsiveness group). Therefore, in high impulsive individuals, some are able to adapt the control strategies to task demands, and some are not. This finding could suggest that the absence of a spontaneous adaptation of control strategies to external demands may be a vulnerability factor for the development of maladaptive behavioral tendencies, reflecting the dysfunctional aspect of impulsivity in a limited number of individuals^27^. Future studies should explore the predictive value of a smaller dominance of proactive control and the inability to adapt the default state of cognitive control, on the emergence of maladaptive behaviors.

The data presented here, along with those previously reported^19^, demonstrate that healthy young individuals are able to adapt control strategies during the AX-CPT task to lean more on proactive processes. One aim of this study was to identify which pattern presented in Fig. 1 explained this adaptation effect by analyzing the RT changes in AY and BX trials across blocks. Results revealed that the increase in the PBI was due to the decrease in reactive control processes, as observed in the decrease in RTs in BX trials (Figs. 1B and 4C). Therefore, the increase in proactive dominance is not due to the increased automaticity of proactive control^19^, but to the decline of effect of reactive control processes. This finding defines the difficulty in control strategy adaptation experienced by the high impulsive individuals. Indeed, the slow adaptation of control processes in the high impulsiveness group seemed to be due to a smaller decrease in reactive processes across blocks compared to that observed in the low impulsiveness group (cf. Fig. 4C). Therefore, impulsive personality traits in a healthy population could be associated with a more reactive control system, and not to a less proactive one. Nevertheless, given that this result was only close to significance, the interpretation of the slower adaptation of control strategies associated with high impulsivity should be taken with caution.

The issue of the multidimensional nature of impulsivity cannot be entirely addressed in the current study. Indeed, the UPPS questionnaire is a self-reported tool to describe an individual’s level of impulsive tendencies following four distinct impulsive dimensions (i.e., *Urgency*, *Premeditation* (lack of), *Perseveration* (lack of) and *Sensation Seeking*). However, the impulsivity construct also comprises behavioral and cognitive components, which can be divided into impulsive action and impulsive decision-making^28,29^. These components are not assessed in the current study. It is thought that these multiple components are only weakly, or not at all, correlated with each other^29–31^. Exploring the relationship between control strategies with the AX-CPT task and additional behavioral measures might be of interest in considering the potential for weaker dominance of proactive control to be used as an overall marker of impulsivity. Indeed, if the pattern of results presented in our study is replicated with other measures of impulsivity, weaker proactive control could be used to define impulsive behavioral strategy^27^. The concept of impulsivity would then encompass all behaviors and traits underpinned by a reduction in the dominance of proactive control. On the contrary, a weaker proactive control could be specific to only a few components of the impulsivity construct. In the current study, we observed significant correlations between the PBI and two specific subscores: *Sensation Seeking* and lack of *Premeditation*. Thus, the present findings favor the second hypothesis and furthermore, confirms previous results reported by Sharif-Razi and collaborators who showed that only the lack of *Premeditation* was related to proactive control difficulties in gamblers^24^. Moreover, numerous studies have shown that some impulsive pathological populations, such as BPD and ADHD, are characterized by distinct impulsive profiles, through their association with different impulsive dimensions^32,33^. Interestingly, these two different impulsive profiles have also been associated with distinct degrees of decreased proactive control dominance^7^. Taking into account the literature and our findings, one hypothesis could be that the reduced dominance of proactive control is common to all impulsive individuals, but is attenuated (or emphasized) according to the nature of the impulsive behavior. Today, studies with larger sample sizes are needed to investigate more precisely the subdimensions of impulsivity in relation to changes in the control strategies through time.

In conclusion, we investigated the dual mechanisms of cognitive control in relation with global impulsive personality traits in the general population. On the one hand, the results revealed that high impulsiveness in the general population was characterized by a less dominant proactive control. On the other hand, we showed that among high impulsive individuals some are unable to spontaneously adapt cognitive control strategies to proactive external demands, potentially explaining the emergence of dysfunctional impulsive behaviors. To go further on this topic, future studies are needed to explore the relationship between cognitive control strategies and the multidimensional nature of impulsivity to differentiate profiles of pathological populations characterized by high impulsiveness. Our work emphasizes the importance to investigate concurrently both the default state and the adaptation of control strategies through time as complementary indices to better understand cognitive control and its association with maladaptive behaviors.

## Methods

### Participants

A total of 48 volunteers recruited in the University of Lille participated in the study (31 women, mean age = 22 years, range from 18 to 39). The sample size was determined based on related studies with similar sample sizes investigating inter-individual differences in control strategies^34,35^. Exclusion criteria included any motor, sensory, psychiatric and/or neurological disorders and a current medical treatment that could affect task performance. Informed consent was obtained from all participants prior to their participation to the study. Ethical approval was obtained from the institutional board of ethics of the University of Lille (2019–341-S70). The experimental was performed in accordance with national and institutional relevant guidelines.

### Procedure and task

#### Task and stimuli

**Figure 6:**
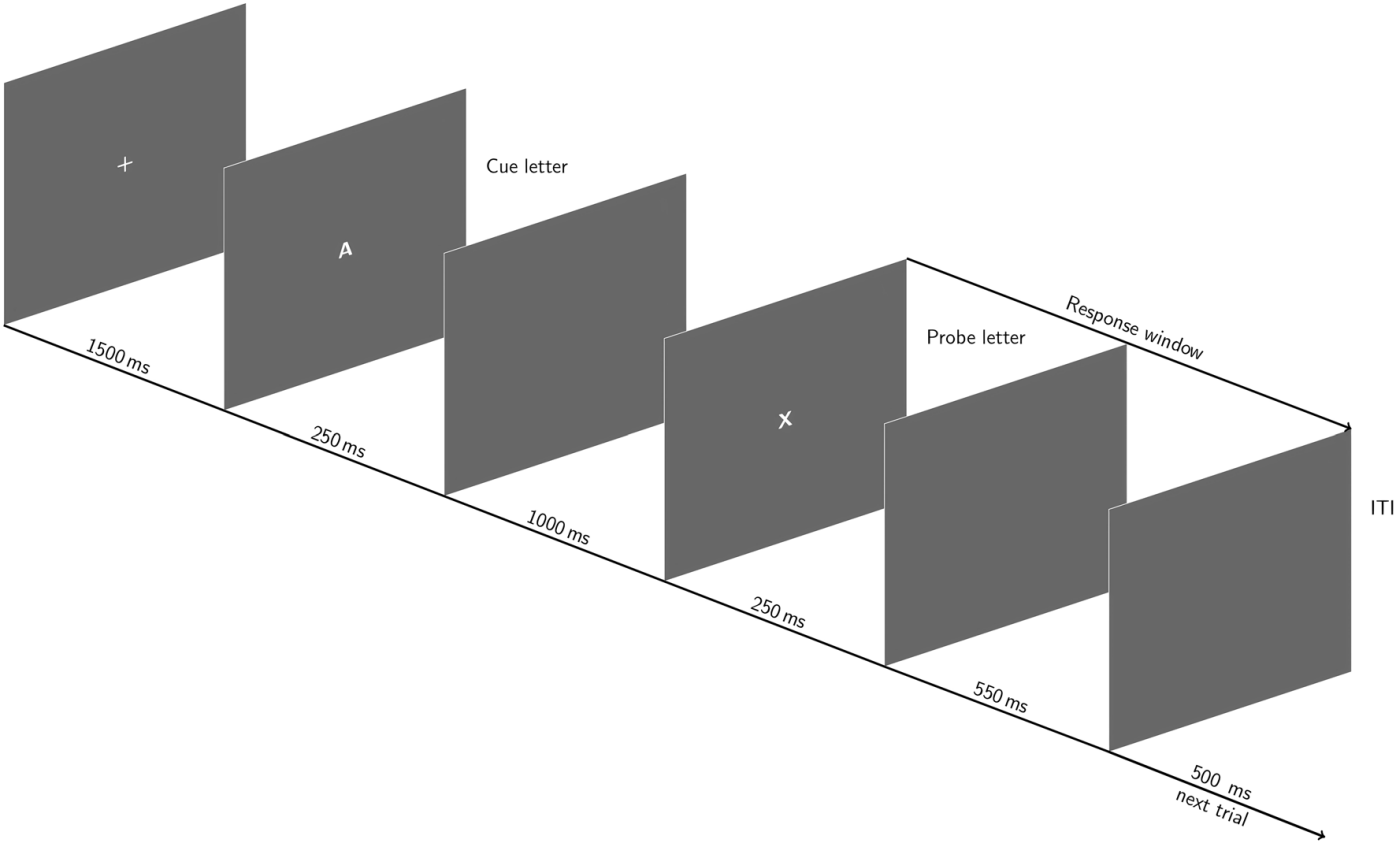
AX-CPT paradigm procedure used in the current study. The figure represents an "AX" trial, which appears in 70% of the trials, and that requires a right response for half the sample. The participant had 800 ms after the probe presentation to respond.

The participant performed the AX-continuous performance task (AX-CPT)^5,6^ implemented using E-Prime experimental software (Psychology Software Tools, Inc.). He/she was invited to respond as quickly and accurately as possible as a function of pairs of letters composed with a cue-letter (i.e., the first letter) and a probe-letter (i.e., the second letter). He/she had to press a response button with the right hand if he/she saw a probe-X only if it was preceded by a cue-A. When the cue-letter was not an A (i.e., generic name “B”) or when the probe-letter was not an X (i.e., generic name “Y”), he/she had to press with the left hand (cf. Fig. 6). All letters were used for the cue-B and the probe-Y letters, excepted for K and Y because of their visual similarity with the X. To ensure the predominance of the response to the cue-A and the probe-X, 70% of the trials were “AX” trials. The other three types of trials (i.e., AY, BX and BY) were each presented in 10% of the trials.

#### Personality questionnaire

The UPPS^18,36^ is a 45-item questionnaire that assesses predispositions for impulsive actions. In our sample, the internal consistency of the UPPS total score was adequate, *α* = 0.90, 95%CI [0.85–0.94]. The median-split method was used to create high and low impulsiveness groups. Participants with a UPPS score below 99 were considered as low impulsive (LI), whereas participants with a UPPS score above 99 were considered as high impulsive (HI). Two participants with UPPS scores equal to 99 were not used in the statistical analysis (N = 46). With an exploratory approach, we also investigated the relationship between cognitive control strategies and impulsiveness through the four UPPS subscales: *Urgency*, *Premeditation* (the lack of), *Perseverance* (the lack of) and *Sensation Seeking*. The *Urgency* subscale refers to the tendency to commit rash and regrettable actions as a result of intense negative affect. The lack of *Premeditation* is the tendency to not carefully think and plan actions. The lack of *Perseverance* corresponds to the inability to remain on a task until completion. Finally, the *Sensation Seeking* scale assesses the tendency to seek adventures and excitement.

#### Experimental procedure

The participant sat in a closed room facing a computer screen. Each trial began with the presentation of a fixation cross at the center of the screen during 1500 ms. The letters were displayed on the center of the screen during 250 ms and were separated by an empty screen for 1000 ms. The participant had 800 ms to respond after the onset of the probe-letter. Then, an empty screen was presented during 500 ms before the start of the next trial. Figure 6 represents the implementation of the task. The experiment began with a training block of 20 trials. During this training, visual feedback appeared for 500 ms after each response providing information about the accuracy of the current trial (*"Bonne réponse"* for a correct response, *"Mauvaise réponse"* for an error, *"Aucune réponse enregistrée"* for responses prior to the probe or responses slower than 800 ms). If at least 90% of the training trials were correct, then the experimental part began. The participant performed 10 blocks of 70 trials. A pause was implemented between each block. The experiment lasted about 50 min.

### Experimental groups and statistical analyses

Reaction times shorter than 50 ms were excluded from the analysis. Then, a 2*SD interval filter was computed on the remaining RTs to eliminate potential performance outliers. One participant was excluded from statistical analysis because of more than 30% of omissions in at least one of the four trial types (*N* = 45). Accuracy rates and mean RTs in correct trials were calculated for each of the four trial types (i.e., AX, AY, BX and BY) and for each participant. Following Braver et al. (2009) methodology^5^, the proactive behavioral index (PBI) was calculated for correct RTs as follows:

$$ PBI = \frac{AY - BX}{{AY + BX}} $$

All the statistical analyses were performed using the *stats* package in RStudio^37^. The graphical representations of the results were performed using the *ggplot2* package^38^. The classical effects of an AX-CPT task were investigated with two one-way ANOVAs with Trial Type (4) as a within-subject factor on accuracy rates and on RTs in correct trials. The ability to adapt behaviors to task demands was investigated by applying a one-way ANOVA with Blocks (10) as a within-subject factor on the PBIs. For some blocks, the PBI could not be calculated as there were only errors and/or omissions in all seven AY and/or BX trials. Five or more non-exploitable PBI led to the exclusion of one participant from this mixed-ANOVA analysis (*N* = 44). Moreover, to investigate the three hypothetical patterns of results presented in Fig. 1, a two-way ANOVA with Blocks (10) and Trials (2) as within-subject factors was performed on mean RTs in correct AY and BX trials.

The effect of impulsiveness on the proactive behavioral index (PBI) was investigated through three distinct analyses. We first analyzed the linear relationship between the UPPS and the PBI using a Pearson’s correlation. Then, we compared the PBI between the high and low impulsiveness groups using a Student *t*-test. Finally, to investigate the evolution of the PBI as a function of blocks and impulsiveness, we performed a two-way mixed-ANOVA with Blocks (10) as within-subject factor and Impulsiveness (2) as a between-subject factor on the PBI.

Additionally, we investigated the effect of impulsiveness on the adaptation of control strategy through the time course of the task. To do so, we computed the estimates of the linear regression model explaining the PBI as a function of Blocks as an index of the adaptation ability. We extracted the model estimate for each individual. The greater the estimate, the stronger is the increase in the PBI across blocks. First, we investigated the linear relationship between the UPPS scores and the estimates using a Pearson’s correlation. Then, considering that a positive estimate indicates a PBI increase (i.e., an adaptation to task demands) and a negative estimate indicates a PBI decrease across blocks (i.e., no adaptation to task demands), we measured the contingency of adaptation as a function of impulsiveness groups using an independence *χ*^2^ test.

Finally, the data were examined by calculating Bayesian statistics with default prior scales, using the *BayesFactor* package^39^. We reported the Bayesian factors (BF). For ease of reading, BF_10_ was used to indicate the BF as evidence in favor of the alternative hypothesis against the null hypothesis.

## Data Availability

The datasets generated during and/or analyzed during the current study are available from the corresponding author on reasonable request.
